# Treatment of Pyorrhea a Specialty

**Published:** 1917-11

**Authors:** F. C. Pague


### TREATMENT OF PYORRHEA A SPECIALTY

BY F. C. PAGUE, D.D.S.

I believe that the treatment of pyorrhea and oral prophylaxis should be regarded as a specialty of dentistry in the same sense that orthodontia is. Fifteen years ago the orthodontist was having the same experience in establishing himself as a specialist as the periodontist is today. The general practitioner is loath to relinquish any work that he can see a possible fee in, and today we occasionally find the general practitioner endeavoring to correct some irregularity when it would be more creditable to him to refer the patient to a specialist. So in pyorrhea, as a dentist said to me once, "You know we are all doing that work now." We find many dentists in general practice trying to treat these conditions, and take a chance in retaining the patient rather than refer him to a specialist qualified to cure him, save the teeth and keep the respect of the patient. Many people are willing to be hoodwinked once, but there are a few who are willing to accept gold bricks all the time, and just as it has worked out for the orthodontist so it will work out for the pyorrhea specialist, and when I say specialist, I mean the dentist confining himself *exclusively* to that work.

To obtain satisfactory results in the treatment of pyorrhea it is absolutely necessary to restore the occlusal surfaces and contact points, to make bridges and removable bridgework and other appliances such as splints, but the pyorrhea specialist can not do this work. The technic of this work, treatment of pyorrhea, is such that not all dentists are qualified to do it, just as physicians are not all qualified to be surgeons, nor all musicians to be leaders. It requires a peculiar temperament, one who is highly sensitive and responsive, to make a success in periodontia.

If the dentist is so constituted that his finger tips will immediately respond to the touch of his keen-edged instrument as it comes in contact with the roughened surfaces on the roots, and he can distinctly detect the minute particles thereon; if he can follow the roots of the anterior teeth to their apices, if the sinus leads thus far, and remove that dense calculus so often to be found there; or can take the multirooted teeth and thoroughly cleanse those bifurcations of their irritating accumulations; if, as I have said, his fingercraft is sufficiently acute to locate and remove these irritants from the roots, then he can surely be classed as one qualified to specialize exclusively in pyorrhea treatment. In the second place, he is not qualified to do the restorative work so necessary for the success of his treatment. The man specializing in pyorrhea can not endure the long, continuous hours of the general practitioner because of the nervous strain; he will lose his sense of touch, and for this reason his hours of operative work must be short, with moments of rest between each patient. The operator with four or five hours of work each day—of say from ten to twelve and two to four, or nine to twelve and two to five—will do far better work than the one confining himself to longer hours.

As the patient of the orthodontist is referred by the general practitioner, or by those interested in patients whom he has treated, just so must the pyorrhea specialist attract his clientele. If the patient requiring subsequent treatment is referred by a dentist, then that patient ought to be referred back to that dentist for whatever work is necessary in correcting malocclusion, restoring contact points, making retaining splints, etc., etc., but all this work ought to be done with the cooperation of the periodontist, that the best results may be obtained. If a patient is referred by one who has been treated by a specialist, then those dentists must be chosen for the subsequent work who are in sympathy with your work, dentists qualified to do this work—and in our larger cities we have such dentists—those who are confining their practice exclusively to the porcelain jacket crown, those confining their practice exclusively to gold inlays, to crown and bridgework; who are in full accord with your judgment—viz., that there should be no overhanging edges, no roughened surfaces, no encroachment on the gums in any sense, from any kind of work whatever, be they fillings of gold or amalgam, the inlay, the gold crown or crowns of any nature.

I believe it is absolutely necessary that we keep the patient in our practice for subsequent prophylactic treatment and if the patient is referred by a dentist he, the dentist, is usually willing that you should keep them, since you are willing to assume the responsibility for a perfectly healthful condition of their mouths, and if the patient comes to you from other recommendation, then, of course, *their* wish will be that you assume the responsibility for subsequent prophylaxis.

Those dentists limiting their practice to periodontia and oral prophylaxis should confine themselves just as exclusively to what they represent as the orthodontist does, and when a dentist represents, by a card or otherwise, that his practice is limited to such work, he should mean that he will not take any part in general practice. When I gave up general practice to confine myself exclusively to this work, I gave up all instruments that would in any way connect me with a general practice. That was the only way I could break away from my practice. I can not see any point for argument as to how or why a dentist specializing in the treatment of pyorrhea can be expected to give his attention to any other work, any more than the orthodontist who is never called upon to extract or place medicine in an aching tooth, and we can only give the best possible service to the public through mutual understanding with the general practitioner, and this we surely can not have, if we are engaged in any work other than oral prophylaxis and periodontia.

I believe the pyorrhea specialist of our larger cities will obtain best results from his work by referring his patients for necessary restorative work to dentists specializing in certain work such as the porcelain jacket crown, and crown and bridgework. If he associates himself with a dentist in general practice who can do this work, he will antagonize the dentist who might refer patients for treatment. Patients referred to me for treatment by the general practitioner are of course referred back to the dentist for whatever restorative work is necessary, and we can work out the necessary detail to the best interest of the patient. Where patients are referred to me by those whom I have treated, I am governed by circumstances, and the restorative work necessary, but generally the patient is referred to a specialist.

In order that we may standardize our own methods of practice, I would like to offer for the consideration of the Academy the following resolutions:

"*Resolved,* That the pyorrhea specialist is one confining himself *exclusively* to the treatment of pyorrhea and oral prophylaxis.

"That the restoring of occlusal surfaces and contact points must be done by the general practitioner.

"That the making of bridges and removable bridgework must be done by the general practitioner or a specialist in such work

"That the making of necessary splints shall be done by the pyorrhea specialist or under his direct observation.

"That we are to keep the patient in our practice for subsequent prophylactic treatment.

"That it is best for us to conduct our practice alone, exclusive of the association of a general practitioner.

"That it would be unwise for us to take into our office a general practitioner to do the work needed in the mouth which we wish for prophylactic reasons to oversee.

"That such an association would affect our relations with the general practitioner."—*Dental Items of Interest* for October.

